# Guanine‐Derived Porous Carbonaceous Materials: Towards C_1_N_1_

**DOI:** 10.1002/cssc.202002274

**Published:** 2020-11-04

**Authors:** Janina Kossmann, Tobias Heil, Markus Antonietti, Nieves López‐Salas

**Affiliations:** ^1^ Colloid Chemistry Department Max Planck Institute of Colloids and Interfaces Am Mühlenberg 1 14476 Potsdam Germany

**Keywords:** basic solid material, carbon dioxide fixation, CN, heterogeneous catalysis, noble carbons

## Abstract

Herein, the basic nature of noble covalent, sp2‐conjugated materials prepared via direct condensation of guanine in the presence of an inorganic salt melt as structure directing agent was studied. At temperatures below 700 °C stable and more basic addition products with at C/N ratio of 1 (C_1_N_1_ adducts) and with rather uniform micropore sizes were formed. Carbonization at higher temperatures broke the structural motif, and N‐doped carbons with 11 wt % and surface areas of 1900 m^2^ g^−1^ were obtained. The capability for CO_2_ sorption and catalytic activity of the materials depended of both their basicity and their pore morphology. The optimization of the synthetic parameters led to very active (100 % conversion) and highly selective (99 % selectivity) heterogeneous base catalysts, as exemplified with the model Knoevenagel condensation of benzaldehyde with malononitrile. The high stability upon oxidation of these covalent materials and their basicity open new perspectives in heterogeneous organocatalysis.

## Graphical abstract

**It's getting hot in here**: Heat treatment of guanine leads to a new material with a C/N ratio of 1.1 (C_1_N_1_). The material shows structural micropores (causing a strong CO_2_ binding), surface area (making them a promising candidate for heterogeneous catalysis), and stability up to 700 °C. At higher temperature, the structure transforms into highly nitrogen‐doped carbons with much larger surface area. This simple direct synthesis results in very promising new materials for several applications like CO_2_ capture and heterogeneous catalysis.

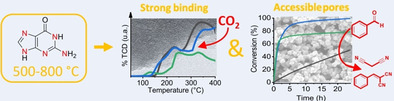

## Introduction

Producing “carbonized” materials has always been considered an uncontrolled process. In 2010 Paraknowitsch et al.^[1]^ and Lee et al.^[2]^ described independently and almost simultaneously the production of carbons from direct pyrolysis of ionic liquids with very special properties (i. e., high temperature resistance under air atmosphere). Later, in 2015, Sakaushi et al.^[3]^ extended the principle to nucleobases, specifically the direct condensation of adenosine. Common to all these new carbons is that the original precursors used were very stable compounds [i. e., that possess a highest occupied molecular orbital (HOMO) level more positive than 1.3 V]. Thus, their carbonization led to even more stable carbonaceous materials due to the bond formation upon carbonization being limited to a number of even more stable motives.^[4]^ Such a hindered bond formation promotes the formation of sp2‐conjugated and layered materials, as aromatic packing is energetically favorable. More interestingly, such stable initial compounds present mostly a high heteroatom content that is usually transferred by a large extent to the final carbonaceous products. Materials fulfilling these conditions were labeled as “noble carbons”.^[4]^

However, the concept of noble carbons is rather new and even the already targeted potential precursors such as nucleobases (e. g., adenine,[^3^, ^5^] guanine,^[6]^ cytosine, or thymine), purines, xanthine derivatives, or ionic liquids^[7]^ have been only roughly explored.

The basicity of heterogeneous materials is classically tuned by surface functionalities. There is however a second option, as the introduction of heteroatoms in the carbon‐π electronic system changes the collective electronic properties and thereby also their acid/base strength.^[8]^ Among the different heteroatoms introduced on carbonaceous networks (e. g., S, N, P, or B), nitrogen is the one that has been most extensively explored by far, due to its capability to facilitate CO_2_ adsorption.^[9]^ Nitrogen doping is normally achieved either by condensation of nitrogen rich organic precursors (e. g., polyaniline, polydopamine) or by post‐treatment of the covalent material (e. g., impregnation of nitrogen‐rich precursors or heat treatment in ammonia atmosphere), while the amount of nitrogen seems to be limited in such thermal “shotgun processes”.^[10]^ Some previous works reported nitrogen contents up to 36 wt % by condensation of nitrogen rich compounds up to 500 °C.[^9a^, ^11^] However, upon increasing the condensation temperature further, the nitrogen content decreased to lower values (e. g., 10 wt % at 1000 °C). Such species seem to be thermodynamically stable and justify the notation “N‐doped carbon”. Therefore, in order to prepare better basic materials in the present context, it is mandatory to control the process and appropriate condensation chemistry at a given temperature.

Carbonaceous materials, or rather high‐temperature organic condensation products, have been the materials of choice for a myriad of applications due to their simple large‐scale synthesis and the versatility of their physico‐chemical properties.^[12]^ Nowadays, gas adsorption and electrochemical energy storage and conversion are among the most common application fields. Interestingly, in 1855, J. Stenhouse already probed that carbon had potential in the field of catalysis, but it was not until the bloom of graphene related materials^[13]^ that carbocatalysis earned well deserved attention.^[14]^ We have recently reviewed the origin of the catalytic activity of carbon materials for those reactions, which otherwise are commonly carried out with transition metals, with the aim to highlight where, in our opinion, carbons have great potential to outperform benchmark catalysts.[^14^, ^15^] Among others, and despite their great potential in chemical synthesis, basic heterogeneous catalysts stand out due to their scarcity. Some reactions like transesterifications, aldol, and Knoevenagel condensation reactions have already been reported to be catalyzed by carbonaceous materials.^[16]^ There, the reaction between malononitrile and benzaldehyde has also been used as a simple way to test the basicity of carbon nitride.^[17]^

Nucleobases not only are ubiquitous, they are Nature's choice to store its most important information. Thus, it is no surprise that they are indeed very stable and as such excellent candidates as precursors towards controlled noble carbonaceous products with high heteroatom contents. Guanine is essentially an oxidized pentamer of HCN^[18]^ and contains equimolar amounts of nitrogen and carbon, and of course it is also easier to work with guanine than with HCN. The molecule as such starts condensing after its melting temperature at 365 °C. As it is oxidized, mostly water and equimolar amounts of CO_2_ and ammonia are produced during condensation, while depolymerization into HCN is suppressed. Herein, we report a more careful analysis of this condensation reaction in terms of the composition of the as‐formed solid‐state organic materials using salt melts as solvents and structure‐directing agent with the aim to show how precoded information in noble carbonaceous precursors leads to the formation of adducts with similar properties and basic character. Guanine was treated at different temperatures using NaCl/ZnCl_2_ (1 : 1) salt mixture. The products obtained were characterized by a variety of local techniques, including thermogravimetric analysis (TGA), Fourier‐transformed infrared (FTIR) spectroscopy, elemental chemical analysis (ECA), scanning and transmission electron microscopy (SEM/TEM), N_2_ and CO_2_ adsorption and desorption isotherms, CO_2_ and temperature programmed desorption (TPD).

We obtained three different classes of products: up to 500 °C, a non‐porous polyguanine was obtained, which transformed into materials with C/N ratio of 1, that is, C_1_N_1_ adducts, with structural porosity that was stable until 700 °C. Further heating to 800 °C resulted in depolymerization and elimination of dicyan and a remaining highly porous N‐doped carbon. The resulting samples were then evaluated in CO_2_ adsorption and as a heterogeneous catalyst for a Knoevenagel condensation reaction, both quantifying the basicity of the materials. It turns out that all condensates up to 700 °C show high basicity, which drops down to ordinary nitrogen basicity for the final N‐doped carbon.

## Results and Discussion

Guanine is an appealing candidate for the production of basic carbonaceous materials due to its high N/C ratio, the presence of amidic functionalities, and precoded potential N‐carbene sites. Guanine as such has under standard conditions 3 acidic/basic sites, p*K*_a_=3.3 (amide), 9.2 (secondary), 12.3 (primary). TGA‐MS of guanine indicates that there is a first mass loss ending at approximately 500 °C, where the major decomposition products are water, carbon dioxide, and ammonia (see Figure S1). As the acidity is vanishing in this step, we have to expect a condensation of the primary amine with the amido group. In that case, the polymer would be left with a rather strong secondary amine. Above 500 °C, more carbon dioxide, water, and ammonia are detached while the carbon‐nitrogen network apparently rearranges and further condenses (see Figure S1). We will see below that this step is combined with the formation of a structural, porous network. Between 700 and 800 °C, massive loss of more heavy molecules, presumably dicyan and its hydrogen adducts, is found, with a final solid‐state residue of approximately 10 wt %. In this process, we have treated guanine within a 1 : 1 NaCl/ZnCl_2_ salt melt (herein called SZ) that acts as a solvent and structure‐directing agent and keeps (by strongly interacting with the precursor) functionalities in the final covalent condensate, thus also leading to higher condensation yields.

A set of bulk samples were then produced by heating guanine up to 500, 600, 700, or 800 °C at 1 °C min^−1^ and then keeping the temperature for 2 h. In order to understand the influence of the dilution on the final product properties, different salt melt ratios (i. e., 1 : 1, 1 : 6 or 1 : 10 guanine/salt melt) were also used. Samples are named hereafter as cG@*t*‐SZ*x*, where *t* stands for the condensation temperature and *x* for the guanine/salt melt ratio. Table 1 shows a summary of the prepared samples and their measured composition [by both energy dispersive X‐ray spectroscopy (EDX) and ECA]. Samples prepared at 500, 600, and 700 °C show a C/N ratio of approximately 1 (similar to that of guanine), independent of the amount of salt used. On the contrary, at 800 °C the C/N ratio raises substantially and by increasing the salt content it changes from 3 to 7. Regarding the samples yield (Table S1), the amount of condense material obtained decreases with increasing temperature, while the yield only weakly depends on the amount of salt. These findings indicate the formation of an amorphous material with at C/N ratio of 1 (C_1_N_1_ adduct) that is stable up to 700 °C and is not altered by dilution in the molten salt solvent throughout condensation. At 800 °C the condensation product has lost its fixed composition, and the yield becomes dependent on dilution. The strong interaction between the salt melt medium and the reacting phase plays here a crucial role stabilizing the leaving functional groups. As a result, the carbonaceous yield increases at higher dilution rates. It is known that ZnCl_2_ can act as dehydrating and condensing catalyst when present in salt melts. To address the contribution of ZnCl_2_ to the formation of C_1_N_1_, a sample was prepared using a ZnCl_2_‐free salt melt (i. e., a 1 : 1 *w*/*w* mix of LiCl and KCl) as template. The obtained material exhibited virtually the same composition as cG@600SZ (i. e., C/N ratio of 1). As can be seen in Table 1, EDX and ECA measured different values for the composition of the samples. The composition found with EDX (measured in high vacuum) looks fair for the bulk samples, while the high oxygen content only found in ECA indicates a bias by massive adsorption under standard conditions (room temperature and standard humidity). We will see below that this is mostly water, which might spontaneously adsorb on the surface of the carbons, that is, the samples act as molecular sieves for water. Moreover, the incomplete mass balance of ECA corroborates the nobility of the samples, as the condensation products are not fully combustible. We leave this observation to the reader as a warning that working with such polar materials always comes with a perturbation of the measurements by adsorbed environmental gases. Standard protocols to clean and degas such porous materials otherwise applied to porous carbons simply cannot be applied to C_1_N_1_ compounds, thus already indicating that we deal with a completely new class of materials.

**Table 1:** Summary of the composition data obtained by EDX, ECA, and ICP analyses and data extracted from physisorption analyses.

Sample	EDX/ECA [wt %]						SSA_BET_^[c]^	*V*_T_^[d]^	Pore diameter^[e]^	CO_2_ adsorption^[f]^
	C	N	O	H^[a]^	Zn^[b]^	C/N	[m^2^ g^−1^]	[cm^3^ g^−1^]	[nm]	[mmol g^−1^]
cG@500‐SZ10	47.7/ 38.0	43.0/ 35.0	3.4/ 14.9	1.6	2.1	1.1/ 1.1	8	–	–	1.6
cG@600‐SZ10	51.7/ 38.0	41.0/ 34.0	7.2/ 17.0	3.0	2.7	1.3/ 1.1	852	0.41 (0.42)	–	3.9
cG@700‐SZ10	46.6	42.4	4.4	–	4.0	1.1	812	0.38 (0.43)	1.2 (1.3)	3.9
cG@800‐SZ10	81.2/ 72.0	11.0/ 12.0	6.7/ 10.8	1.0	0.3	7.4/ 6.5	1982	1.40 (1.42)	4.1 (3.6)–(1.7)	4.7

[a] Only by ECA. [b] From inductively coupled plasma optical emission spectroscopy (ICP‐OES). [c] Specific surface area obtained by applying the Brunauer‐Emmett‐Teller (BET) method to N_2_ adsorption isotherms at 77 K. [d] Total pore volume (*V*_T_ ) calculated by DFT, in parentheses the result obtained at *P*/*P*_0_=0.95. [e] Pore diameter as obtained from QSDT method applied to N_2_ adsorption isotherms at 77 K (in parentheses the values obtained applying the same model to Ar adsorption isotherms at 87 K). [f] Amount of CO_2_ adsorbed at 273 K at 800 torr.

**Figure 1:**
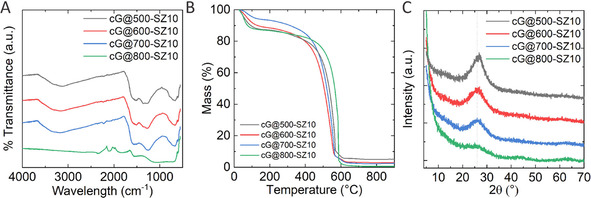
(A) FTIR, (B) TGA traces in synthetic air atmosphere, and (C) XRD pattern of samples prepared at different temperatures.

FTIR as well as Raman spectra of the materials evidence the similarities of samples condensed at 500, 600, and 700 °C (see Figure 1a and Figure S2). FTIR spectra of the materials show a broad peak at approximately 1200–1600 cm^−1^, which can be ascribed to the stretching mode of CN heterocycles, and a broad band in the range between 2800 and 3700 cm^−1^ that can be ascribed to water or deformation and stretching modes of NH vibration. For the 800 °C sample, the FTIR spectra do not show clear peaks anymore, further highlighting the different nature of this sample. Raman spectra of all the samples show very broad peaks, indicating that, if any, the crystalline sites of the samples are very small. The deconvolution of the spectra of samples condensed at 500, 600, and 700 °C show three main peaks centered at approximately 1210, 1360, and 1550 cm^−1^, which can be ascribed to C−N functionalities, and the typical D and G C−C bands. The deconvolution of cG@800‐SZ10 shows that, when the temperature is risen to 800 °C, the G band intensity decreases and a fourth peak at 1495 cm^−1^ appears. This peak is usually ascribed to a D’’ mode of carbon. The X‐ray diffraction (XRD) patterns (Figure 1c) of samples cG@500‐SZ10, cG@600‐SZ10, and cG@700‐SZ10 are again very similar, and the slight decrease of the peak at 26.5° upon increasing the condensation temperature might indicate an increase of amorphous content. The XRD pattern of cG@800‐SZ10 shows virtually no graphitic diffraction peak, which might indicate that at 800 °C the presence of an amorphous phase is dominant. (Figure 1 and Figure S3). The XRD patterns of samples with increasing salt melt (Figure S4) further corroborate this hypothesis. For instance, while increasing the presence of salt melt seems to have no effect on the diffraction pattern of the samples at 500 °C, at 800 °C a constant decrease of the diffraction features is observed.

TGA in synthetic air of the samples showed how the resulting materials resist upon oxidation (Figure 1b and Figure S5), independently of their chemical composition. All samples easily survive 400, if not 500 °C in air, that is, they are noble by character. Here it is important to highlight that all materials where degassed at 150 °C for 20 h under vacuum previous to the TGA experiments, as alerted by the ECA results. Without this pretreatment, a much bigger mass loss at 100 °C due to water adsorbed on the materials was detected by TGA‐MS (Figure S6). The harsh conditions necessary to remove the water in the samples indicate that the materials are able to bind polar hosts strongly.

**Figure 2:**
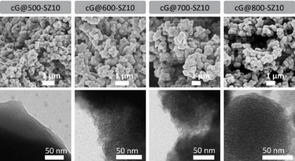
SEM (upper row) and TEM (lower row) micrographs of guanine condensed at different temperatures with guanine/SZ ratio 1 : 10.

**Figure 3:**
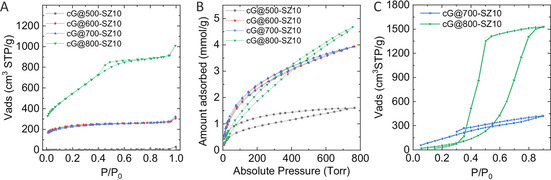
(A) N_2_ adsorption/desorption isotherms at 77 K and (B) CO_2_ adsorption/desorption isotherms at 273 K of samples condensed at different temperatures using a 1 : 10 guanine/salt melt ratio, and (C) water vapour adsorption/desorption isotherms of cG@700‐SZ10 and cG@800‐SZ10.

X‐ray photoelectron spectroscopy (XPS) helps elucidating whether carbon‐nitrogen functionalities suffer dramatic changes upon increasing the temperature. Figure S7 shows the deconvoluted N 1 s and C 1 s XPS spectra of cG@500‐SZ10, cG@600‐SZ10, and cG@800‐SZ10. For all samples, the C 1 s signal is the result of adding up signals from C=C bonds (284.7 eV) and C−N bonds (286.1 eV), and a third deconvolution peak centered at 286.5 eV ascribed to C=O bonds (that can come from both the material itself and adventitious CO_2_). When the temperature rises to 800 °C the ratio between C=C and C−N bonds increases, which is in good agreement with the elemental composition. N 1 s signal deconvolution shows four types of nitrogen functionalities: N‐pyridinic (398.2 eV), N‐pyrrolic (399.7 eV), a very electron‐poor N‐quaternary in plane (400.5 eV) and a broad band 405 eV related to N‐oxide. The ratio of the signals changes when the temperature increases to 800 °C, with an increase of electron‐poor nitrogen signals. The different intensities follow the partial destruction of the C_1_N_1_ adduct. To summarize the XPS results, it can be seen that increasing the condensation temperature shifts the C 1 s signal to lower binding energies and N 1 s to higher ones. As basicity of nitrogen relies on rather electron‐rich nitrogen atoms and is absent in N^+^‐species, we expect larger parts of basicity to be lost in the final depolymerization step. Figure 2 compares SEM and TEM images of samples prepared at different temperatures using a 1 : 10 guanine/salt melt ratio. Micrographs of all series indicate the formation of a xerogel‐like network structure build up by primary particles with a diameter of 300–1000 nm. We assume that these particles precipitate from the original homogenous salt solution once the condensates reach a certain molecular weight in a spinodal process, as it is typical for polymer colloids.^[19]^ SEM shows how all samples exhibit similar structures, whereas the TEM images shows how within these primary spheres porosity develops. We observe the transit from a total absence of any microporous texture at 500 °C to well homogeneous porous materials. EDX elemental mapping of the samples shows that nitrogen is homogenously distributed in the materials at every temperature tested (see Figure S8). All this information suggest that the primary condensate formed at 500 °C slowly rearranges into a more defined porous carbonaceous material up to 700 °C. Then, at 800 °C, we obviously leave the thermodynamic stability range of C_1_N_1_, and the former material depolymerizes and leaves a nitrogen‐doped carbon behind (as observed by XPS). This occurs under some slight shrinking of the primary spheres and the development of a typical carbon micropore structure, as reflected in TEM. Figure S9 displays further micrographs obtained by SEM of samples prepared with increasing guanine to salt melt ratio at both 500 °C (i. e., cG@500‐SZ1, cG@500‐SZ6, and cG@500‐SZ10) and 800 °C (i. e., cG@800‐SZ1, cG@800‐SZ6, and cG@800‐SZ10). The images reveal how in both sets of samples the salt melt is able to induce the formation of a carbon porous network and follow similar trends upon dilution. N_2_ and CO_2_ adsorption/desorption isotherms help to understand the pore morphology and to assess the basic nature of the samples. When keeping a 1 : 10 guanine/salt melt ratio constant and rising the condensation temperature from 500 to 800 °C, samples above 500 °C start developing significant micropore volumes and thus, large surface areas, as predicted by TEM (see N_2_ adsorption/desorption isotherms in Figure 3a). As can be seen, sample cG@500‐SZ10 shows virtually no surface area. Remarkably, both cG@600‐SZ10 and cG@700‐SZ10 exhibit a virtually equal type I N_2_ adsorption‐desorption isotherm. On the other hand, the cG@800‐SZ10 isotherm indicates that the sample not only presents a larger volume of micropores, but also shows a sharp slope at intermediate relative pressures. This might indicate that, while the amorphous content increases, the occurrence of unstacking of graphitic layers happens too. The formation of mesopores would then be the result of the combination of both the rupture of C_1_N_1_ due to the lack of stability at that temperature and the larger evaporation of Zn ions from the salt melt used as solvent and structure‐directing agent. The boiling points of pure ZnCl_2_ and NaCl are 732 and 1461 °C, respectively. However, as revealed by the TGA performed on the reacting mixture, the decrease in mass (i. e., increase vapor pressure of the metal halides) is already significant at lower temperatures as seen in Figure S10, which is further proof that the decomposition of C_1_N_1_ fosters the formation of larger surface areas.^[20]^ These changes provide cG@800‐SZ10 with a 1982 m^2^ g^−1^ specific surface area (SSA), whereas cG@600‐SZ10 and cG@700‐SZ10 present a SSA of approximately 852 m^2^ g^−1^. Such sorption isotherms are similar to COFs and MOFs, but rather untypical for ordinary activated carbons. For instance, the explosion of surface area was already observed for the transition of density‐optimized covalent triazine frameworks (CTFs) to popped‐up, thermally delaminated CTFs.^[21]^ Figure S11a, b gives further sorption data on the effect of temperature and salt amount. The figures show the N_2_ adsorption/desorption isotherms of samples prepared at 500 and 800 °C, respectively, when increasing the amount of salt melt in the synthesis. Remarkably, all isotherms at 500 °C show virtually no surface area while at 800 °C a remarkable increase on the final SSA occurs with increasing amount of salt melt, again confirming the nanoscopic “popcorn” effect. While the composition did not change at 500 °C, the samples 800 °C show a change from 1 : 1 to 1 : 6, and then the composition is maintained though not anymore that C_1_N_1_ in any of the samples. This is further proof that at this temperature the surface area development is the result of the combination of ZnCl_2_ vapor pressure and rupture of C_1_N_1_ (Table S2). These results characterize the three different types of materials throughout temperature increase. At 500 °C a solid with no accessible surface area formed of precipitated guanine‐derived oligomers is obtained. Though it has precipitated out of the salt melt, scaffolding has not yet taken place, and thus no porosity is observed. The materials obtained at 600 and 700 °C are stable C_1_N_1_ porous samples reminiscent of CTFs. As previous results evidenced, at 800 °C C_1_N_1_ is not stable anymore, and a nitrogen‐doped carbon with super high surface area is obtained due to sample destacking and loss of nitrogen at high salt melt dilution. Ar adsorption‐desorption isotherms at 87 K of cG@700‐SZ10 and cG@800‐SZ10 were performed to further understand the microporous nature of the samples. As can be seen in Figure S12, both samples exhibit narrow distributions around 1.3 and 1.7 nm, respectively.

CO_2_ isotherms not only further describe the pore morphology of the samples, but also add information on possible acid‐base interactions. For instance, having a close look at Figure 3b, one can see how CO_2_ adsorption isotherms of the samples at different condensation temperatures change with structural evolution. All the samples above 500 °C are able to adsorb rather similar volumes of CO_2_, but curve shapes indicate how different the CO_2_ adsorption sites in fact are. Samples at 500 °C show a remarkably high CO_2_ sorption despite their virtually null N_2_ adsorption, while strong hysteresis points to the kinetical hindrance throughout the process. C_1_N_1_ oligomeric precipitates obtained at 500 °C are able to take up approximately 1.5 mmol g^−1^ of CO_2_ through very specific sites that are yet not accessible (i. e., hidden) for nitrogen. C_1_N_1_ samples obtained at 600 and 700 °C exhibit the same type of specific structural sites plus the structural micropores that make them accessible to CO_2_ (i. e., not hidden anymore). Thus, they reach an adsorption capacity of up to 4 mmol g^−1^ of CO_2_. Where the shape of the isotherm indicated strong binding at already rather low pressures, the comparison with the internal reference of an N‐doped carbon is indeed most rewarding. In spite of the fact that the micropore volume observed by nitrogen physisorption for sample cG@800‐SZ10 is much larger (i. e., almost double), CO_2_ adsorption on this sample is remarkably weaker and exceeds the values of cG@600‐SZ10 and cG@700‐SZ10 only at higher pressures and even then only by approximately 0.5 mmol g^−1^. This means that the binding of CO_2_ in C_1_N_1_ and N‐doped carbons are fundamentally different, with a much higher binding strength in C_1_N_1_. The 800 °C sample again behaves similar to other highly N‐doped porous carbons, where CO_2_ uptake is mainly due a large micropore volume (i. e., physical CO_2_ sorption). Figure S10c, d presents the CO_2_ adsorption isotherms of samples prepared at 500 °C (i. e., cG@500‐SZ1, cG@500‐SZ6, and cG@500‐SZ10) and 800 °C (i. e., cG@800‐SZ1, cG@800‐SZ6, and cG@800‐SZ10) with different salt melt ratios. Samples made at 800 °C show the same CO_2_ sorption capability independent of the differences in their surface area and apparent micropore volumes, as determined by their N_2_ isotherms. Water vapor physisorption (Figure 3C) reinforces the strong water adsorption assumed by ECA and TGA. cG@800‐SZ10 shows a remarkable high water uptake of around 1500 cm^3^ g^−1^ at high pressure, proving the highly polar character of the material. Compared to cg@800‐SZ10, the water uptake of cG@700‐SZ10 seems not as remarkable, but compared to other carbonaceous materials, an uptake of approximately 420 cm^3^ g^−1^ at high pressures is still among the higher values reported up to date.^[22]^ Moreover, the incomplete desorption of cG@700‐SZ10 proves the strong binding of water to the polar surface as predicted by elemental analysis.

**Figure 4:**
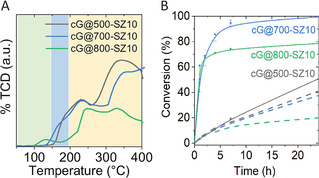
(A) CO_2_ TPD traces obtained using cG@500‐SZ10, cG@700‐SZ10, and cG@800‐SZ10. Green area represents CO_2_ physisorbed in micropores, blue area represents CO_2_ bonded to highly nitrogen‐doped pores, and yellow area is ascribed to CO_2_ bonded to structural C_1_N_1_ sites. (B) Conversion vs. time as catalyst for the Knoevenagel condensation reaction of benzaldehyde with malononitrile. Solid lines stand for materials used after degassing at 150 °C for 20 h and dashed lines stands for materials used without previous degassing.

CO_2_ TPD measurements displayed in Figure 4a further corroborate the different interaction of the three types of samples with CO_2_ (i. e., cG@500‐SZ10, cG@700‐SZ10, and cG@800‐SZ10). Here, it needs to be stated that the samples are loaded at ambient temperatures and 1 bar of CO_2_, that is, conditions where most activated carbons already only partly bind CO_2_ at their surface. The most intense signal is found for sample cG@500‐SZ10. The TPD trace shows two CO_2_ desorption broad peaks centered at 200 and 330 °C and no peaks up to 100 °C, temperatures up to which physisorbed CO_2_ would de‐bound. This indicates that CO_2_ binds to the material through two types of sites that possess very high binding energies, well beyond physisorption. Here, it is important to remember that the sample exhibits an extremely low Brunauer‐Emmett‐Teller (BET) surface area, but still a surprisingly high CO_2_ binding capability of 1.5 mmol g^−1^.

Sample cG@700‐SZ10 shows a similar CO_2_ TPD signal to that of cG@500‐SZ10, but a shoulder develops at temperatures ranging from 150 to 200 °C, which we attribute to the coexistence of some larger micropores. This sample has a significantly larger SSA and a significant volume of micropores which goes along with the capability of the sample to adsorb up to 4 mmol g^−1^ of CO_2_ at room temperature. However, the TPD shape indicates that the strong structural binding sites remain until 700 °C. In the TPD trace of cG@800‐SZ10 (with even larger SSA), a new peak at ca. 130 °C appears (typically ascribed to physisorption of CO_2_ on a noble carbon^[23]^) while the peaks at higher temperatures are significantly lower in intensity. This strongly indicates that at 800 °C only some of the strong binding sites remain, but that most of the CO_2_ adsorption is either invisible in TPD, or remarkably weaker. Nevertheless, even this sample still shows a very good CO_2_ binding capability, especially when compared to standard activated carbons.

**Figure 5:**
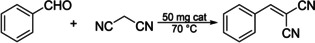
Knoevenagel reaction followed in this paper.

To analyze the basic character of cG@500‐SZ10, cG@700‐SZ10, and cG@800‐SZ10 as heterogeneous catalysts, the Knoevenagel condensation reaction of benzaldehyde with malononitrile was selected as a model reaction (see Figure 5). The values of conversion of benzaldehyde and malononitrile to 2‐benzylidenemalononitrile using the different materials as catalysts are shown in Figure 4b. Samples were degassed at 150 °C for more than 20 h under high vacuum previous to each experiment to ensure that the active sites of the materials are fully available for the reactants, as the materials spontaneously bind water and CO_2_ at ambient conditions. After this de‐binding step, all catalysts were more active and show 99 % selectivity towards 2‐benzylidenemalononitrile. The sample condensed at 700 °C shows the highest conversion of 99 % after 24 h with 68 % after 2 h. cG@800‐SZ10 also reaches 67 % conversion after 2 h. However, within the next 20 h the conversion reaches up to only 78 %. cG@500‐SZ10, which showed the most intense CO_2_ TPD trace, exhibits slower kinetics. After 2 h conversion reaches values under 10 % and within the next 20 h it slowly increases up to 50 %. The lower activity of cG@500‐SZ10 is ascribed to its almost non‐existing SSA, which makes the active sites unreachable for the substrates, much larger than CO_2_. Additional different substrates were tested to investigate the influence of substitution of the aldehyde to the reactivity using cG@700‐SZ10 as catalyst. Substitution of benzaldehyde with chloride or methyl groups in position 4 lowers the conversion to 85 and 68 %, respectively, which can be ascribed to the increasing size of the substrate. Using furfural instead of benzaldehyde further reduces the conversion to 46 %, which can be explained by the change in the polarity induced by the furan ring.

The majority of heterogeneous base catalysts in the literature are pre‐treated with strong bases to activate the base functionalities (to de‐bind potentially pre‐bounded acids), while simple degassing works for guanine‐derived porous condensates (see Table S2).[^17^, ^24^] The results indicate that the design of basic catalysts strongly depends on both their composition and pore morphology, balancing the effects of activation, activity, and accessibility. Precoding information using noble organic precursor is possible and offers great possibilities, since the final catalyst not only could be reused by simple heat treatments, but their high thermal and oxidation stability allows their potential use in high‐temperature catalytic reactions that are currently not accessible by other covalent materials.

## Conclusions

A series of noble covalent materials were prepared using guanine as precursor and salt melts as solvent and structure‐directing agent. Three different types of materials with very different properties were obtained by increasing the thermal condensation temperature. At 500 °C oligomers with C/N ratio of 1 (C_1_N_1_) precipitate out of the salt melt phase. By raising the temperature up to 700 °C C_1_N_1_ adducts with well defined microporosity are formed. Chemical analysis of the samples indicates that dilution in salt melts does not affect C_1_N_1_ at temperatures below 700 °C. However, at 800 °C, stability of the adducts is lost, and popped‐up, high‐surface‐area, noble, N‐doped carbonaceous materials with a C_7_N composition are obtained. Sorption experiments indicate that the structural C_1_N_1_ pores bind CO_2_ with a strength previously not known from covalent organic materials. The basicity of these structural pores was proven by using the materials as organocatalysts, and their performance was evaluated for the Knoevenagel reaction between malononitrile and benzaldehyde. Conversions and selectivities exceeding 99 % could be reached, which excels above previously reported catalysts that needed activation treatments to de‐bind acidic species. The sample stability and tunability opens the way to utilize these new covalent materials in highly oxidative or high‐temperature catalytic reactions that are currently not covered by benchmark solid organic catalysts.

## Experimental Section

**Materials**: Guanine 99 % (G), sodium chloride (99.5 %), zinc chloride, acetonitrile, and benzaldehyde were purchased from Merck. Malonitrile was purchased from Acros Organics, and all materials were used as received.

**Synthesis**: The salt mixture was freshly prepared prior to each synthesis by grinding NaCl and ZnCl_2_ in a 1 : 1 ratio (*w*/*w*) with a pestle and a mortar (melting point: 325 °C). Covalent networks were prepared by simply mixing guanine (1 g) with different amounts of the salt mixture (i. e., 1, 6, or 10 g) (see Table S1). All together were put into a ceramic crucible with a ceramic cap and condensed under N_2_ atmosphere for 2 h at different temperatures ranging from 500 to 800 °C using a 1 °C min^−1^ heating ramp. After cooling down the samples were washed in 300 mL 1 m HCl three times and dried at 70 °C for 3 h.

**Catalytic test**: Catalytic activity of the as prepared materials was tested for the Knoevenagel condensation reaction of benzaldehyde with malononitrile. In a typical reaction, 50 mg of catalyst was added to 10 mL of acetonitrile solution containing 1 mmol of aldehyde and 2 mmol of malononitrile (or ethylcyanacetate). The reaction mixtures were stirred at 70 °C for different times in an oil bath. Samples were evaluated using ^1^H NMR spectroscopy and GC‐MS analysis. The product was identified by using an offline GC with an HP‐5MS column (inner diameter=0.25 mm, length=30 m, film=0.25 μm) with a MS (Agilent GC 6890, Agilent MSD 5975). The percentage conversion was calculated by ^1^H NMR spectroscopy, recorded on Agilent 400 MHz.

**Characterization**: TGA was performed from 25 to 1000 °C in a NETZSCHTG 209 F1 device using either nitrogen or synthetic air as carrier gas and a heating rate of 10 K min^−1^ in a Pt crucible. TGA‐MS helped elucidating the products of thermal decomposition of the initial precursors and the produced nucleobases. TGA‐MS measurements were performed using a thermo microbalance TG 209 F1 Libra (Netzsch, Selb, Germany) coupled with a Thermostar MS (Pfeiffer Vacuum; Asslar/Germany) with a ionization energy of 75 eV. A platinum crucible was used for the measurement of 10 mg of the samples. Samples were heated at 2.5 K min^−1^ to 910 °C in a helium flow of 10 mL min^−1^ and a purge flow of 10 mL min^−1^. Data were recorded and analyzed by the Proteus (6.0.0) and Quadstar (7.03, MID modus) software package. ECA was carried out using a vario MICRO cube CHNOS Elemental Analyzer (Elementar Analysensysteme GmbH, Langenselbold) in the CHNS mode and a 2mgChem80s Method. Morphology of the samples was analyzed by SEM using a Zeiss Gemini 1550 microscope. To improve surface conductivity, an approximately 10 nm thick film of an 80 % gold/20 % palladium alloy was sputtered on top of the sample prior investigation. EDX was acquired using a coupled Oxford Instruments EDX analyzer. TEM was carried out with a Zeiss 912 Omega instrument, scanning transmission electron microscopy (STEM) was carried out with a double‐Cs‐corrected Jeol ARM200F instrument. Powder XRD patterns were recorded using a Bruker D8 Advance instrument with CuK_α_ radiation. Prior to each physisorption measurement samples were degassed for 20 h at 150 °C. Nitrogen adsorption and desorption isotherms at 77 K and CO_2_ adsorption and desorption isotherms at 273 K (ice‐water bath) were measured using a Quantachrome Quadrasorb SI apparatus. The specific surface area of each material was obtained from the nitrogen adsorption data (*P*/*P*_0_<0.2) using the BET method with the Rouquerol criteria. The value was obtained after applying the method in the linear region with the best correlation. Argon physisorption at 87 K and water vapor physisorption at 298 K were performed on an Autosorb IQ (Quantachrome Instrument). The pore size distributions were obtained by applying Quenched Solid Density Functional Theory (QSDFT) model with slit/cylindrical pore shape using argon adsorption data at 87 K. FTIR spectra were recorded on Thermo Scientific Nicolet iD5 spectrometer. XPS measurements were performed on a Thermo Fisher Scientific Escalab 250 Xi. CO_2_‐TPD was measured using a Micromeritics Autochem II 2920 analyzer. The sample (0.1 g) in a quartz microreactor was pretreated with He (40 cm^3^ min^−1^) at 423 K for 60 min, cooled down to 323 K and the CO_2_ chemisorption were taken place in 20 consecutive pulses (25 vol % CO_2_/He mixture, 1 cm^3^ STP per pulse). After purging with He (10 cm^3^ STP min^−1^) for 90 min, desorption was monitored until 673 K with a heating rate of 10 K min^−1^ and a He flow of 25 cm^3^ STP min^−1^.^[25]^

## Conflict of interest

The authors declare no conflict of interest.

## Supporting information

As a service to our authors and readers, this journal provides supporting information supplied by the authors. Such materials are peer reviewed and may be re‐organized for online delivery, but are not copy‐edited or typeset. Technical support issues arising from supporting information (other than missing files) should be addressed to the authors.

Supplementary
