# Supplementary material for: Guanine‐Derived Porous Carbonaceous Materials: Towards C1N1
Source: ChemSusChem. 2020 Nov 4;13(24):6643–50. doi: 10.1002/cssc.202002274 (PMC7756593; doi:10.1002/cssc.202002274)
Supplement: Supplementary file 1 — Supplementary [file CSSC-13-6643-s001.pdf]

ChemSusChem

Supporting Information

Guanine-Derived Porous Carbonaceous Materials: Towards C_1N_1

Janina Kossmann, Tobias Heil, Markus Antonietti, and Nieves López-Salas*© 2020 The Authors. ChemSusChem published by Wiley-VCH GmbH. This is an open access article under the terms of the Creative Commons Attribution License, which permits use, distribution and reproduction in any medium, provided the original work is properly cited.

Table S1. Synthetic details.

Entry	Sample	Guanine /g	$NaCl/ZnCl_2$ / g	Temp. treatment program	Carbon yield (%)
1	cG@500-SZ1	1	1	RT to 500 at 1 °C/min, 2 h at 500°C	60
2	cG@500-SZ6	1	6	RT to 500 at 1 °C/min, 2 h at 500°C	64
3	cG@500-SZ10	1	10	RT to 500 at 1 °C/min, 2 h at 500°C	61
4	cG@600-SZ10	1	10	RT to 600 at 1 °C/min, 2 h at 600°C	42
5	cG@700-SZ10	1	10	RT to 700 at 1 °C/min, 2 h at 700°C	33
6	cG@800-SZ1	1	1	RT to 800 at 1 °C/min, 2 h at 800°C	16
7	cG@800-SZ6	1	6	RT to 800 at 1 °C/min, 2 h at 800°C	20
8	cG@800-SZ10	1	10	RT to 800 at 1 °C/min, 2 h at 800°C	22
9	cG@800-SZ15	1	15	RT to 800 at 1 °C/min, 2 h at 800°C	23

Figure S1. Different ion currents measured by MS during thermogravimetric analysis of guanine in N_2 atmosphere.

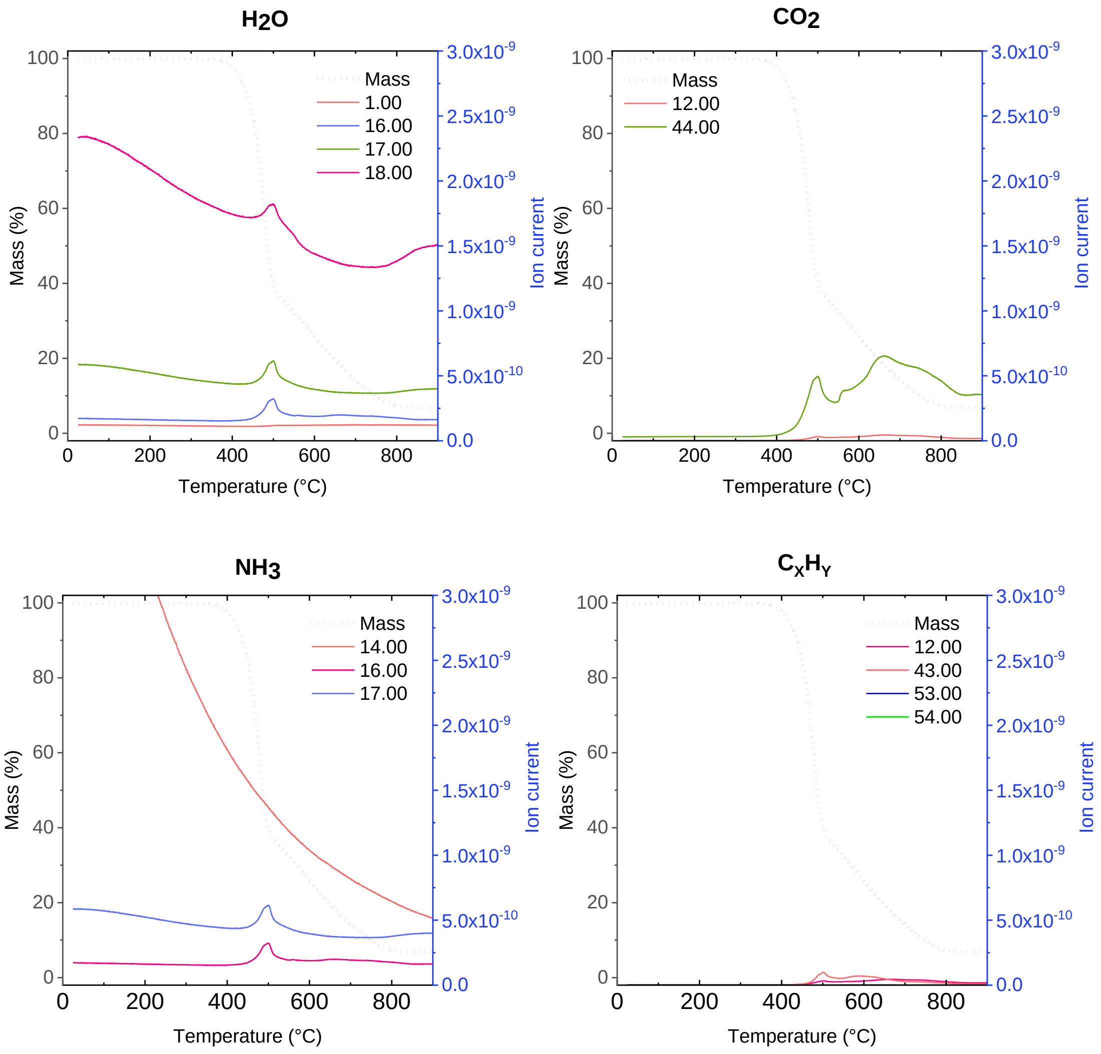

Figure S2. Raman spectra of cG500-SZ10 (A), cG@600-SZ10 (B). cG@700-SZ10 (C) and cG@800-SZ10 (D) with peak deconvolution using Quadratic Savitzky-Golay method.

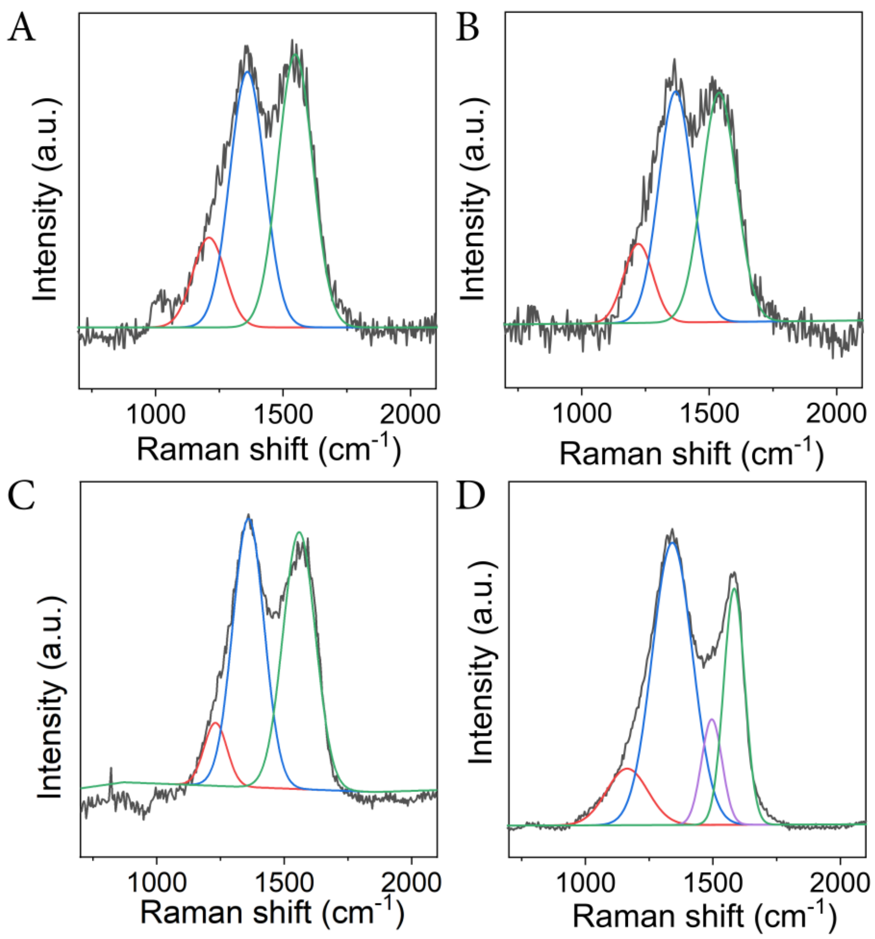

Figure S3. Bright field (upper row) and dark field (lower row) STEM micrographs of cG@800-SZ10 with different magnifications**.**

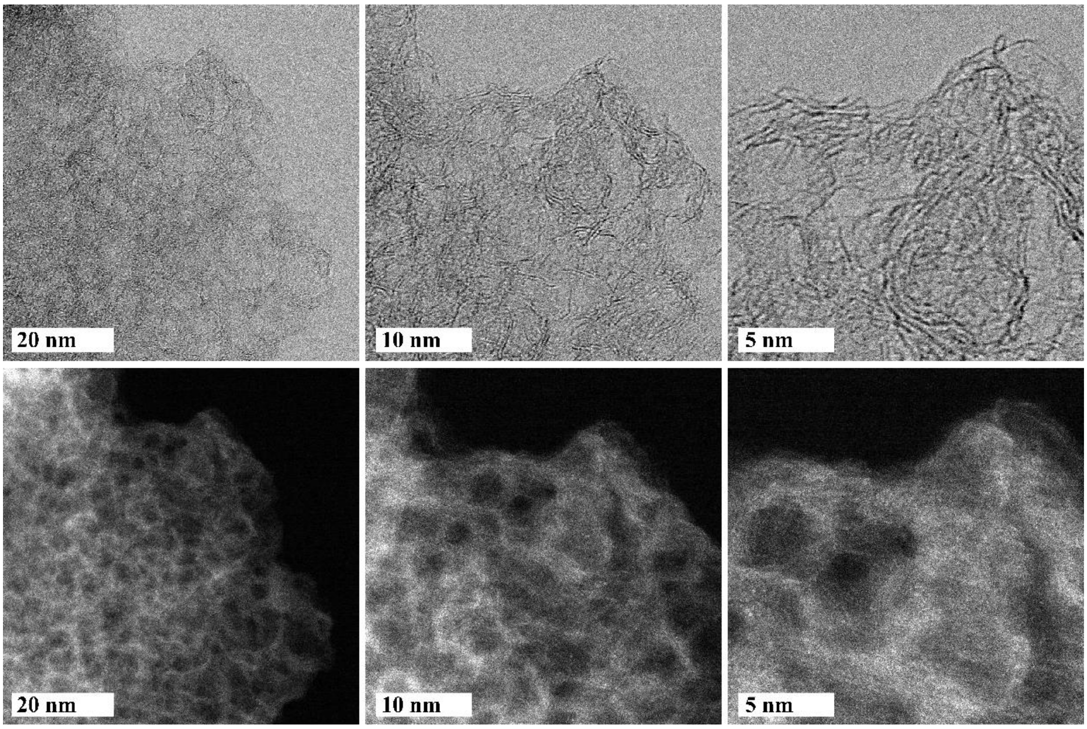

Figure S4. XRD pattern of guanine heat-treated at 500 °C (A) and 800 °C (B) with different precursor to porogen ratio.

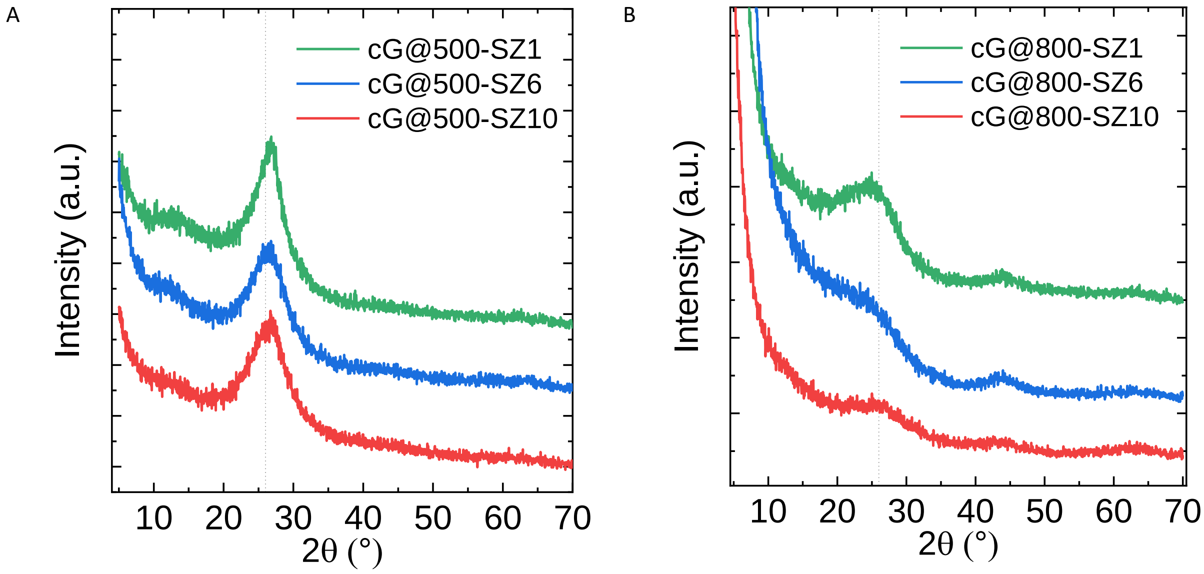

Figure S5. TGA under synthetic air atmosphere of guanine condensed at 500 °C (A) and 800 °C (B) with different porogen to precursor ratios and condensed at different temperatures with a guanine to precursor ratio of 1:10 before activation (C).

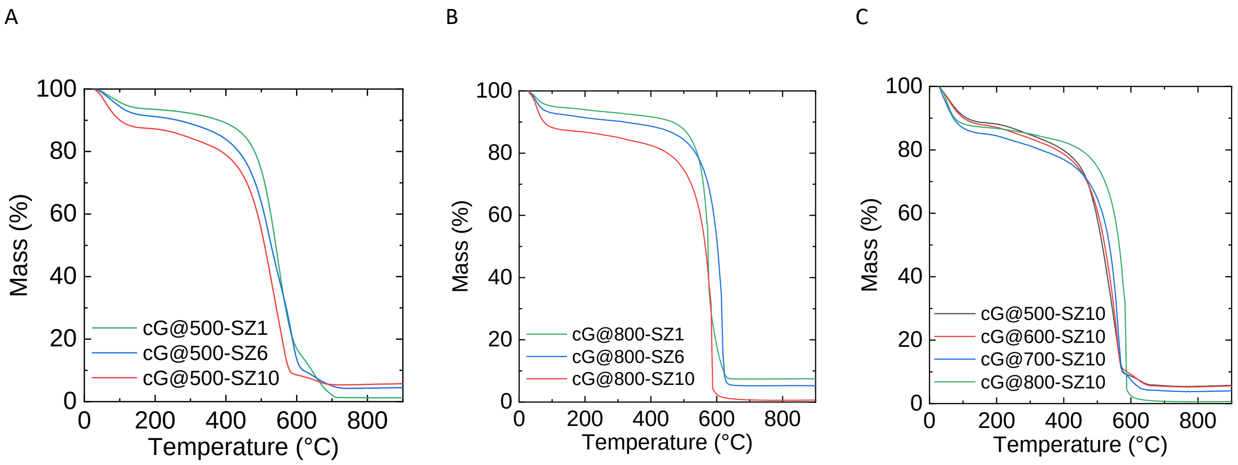

Figure S6. Water related ion currents detected by TGA-MS during thermogravimetric analysis of cG@500-SZ10 (A), cG@600-SZ10 (B), cG@700-SZ10 (C), and cG@800-SZ10 (D).

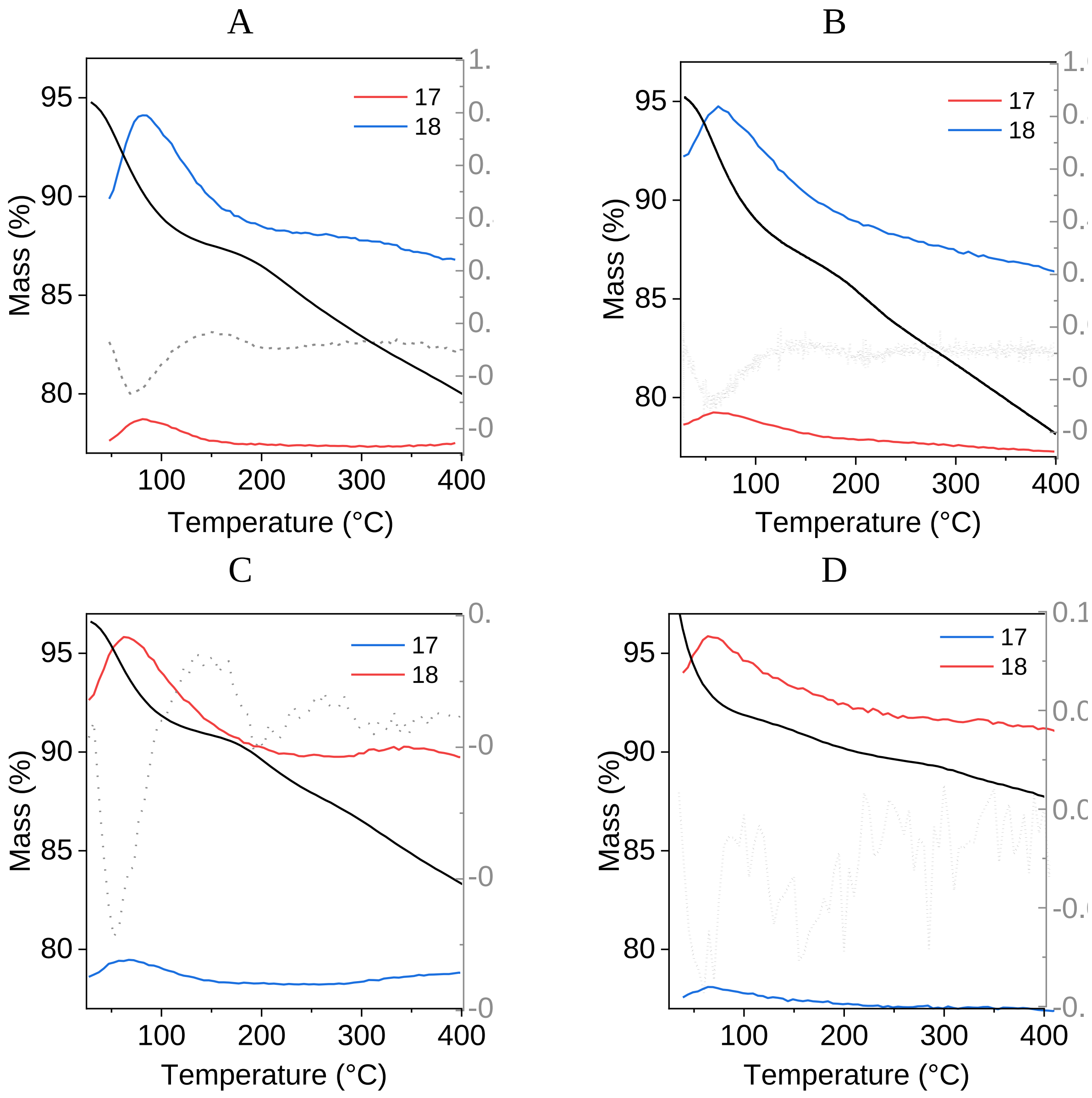

Figure S7. C1s, N1s, and O1s convoluted peaks of samples cG@500-SZ10, cG@700-SZ10, and cG@800-SZ10.

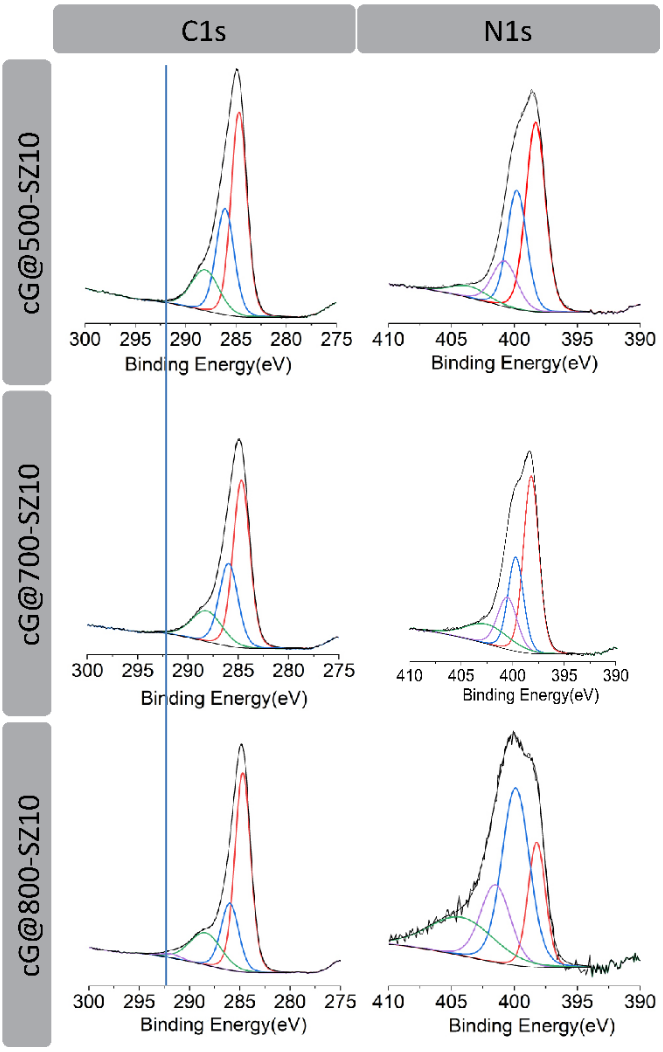

Figure S8. SEM micrograph and EDX elemental mapping of guanine condensed at 500 °C, 600 °C, 700 °C, and 800 °C with a guanine to porogen ratio of 1:10. All scale bars: 10 nm.

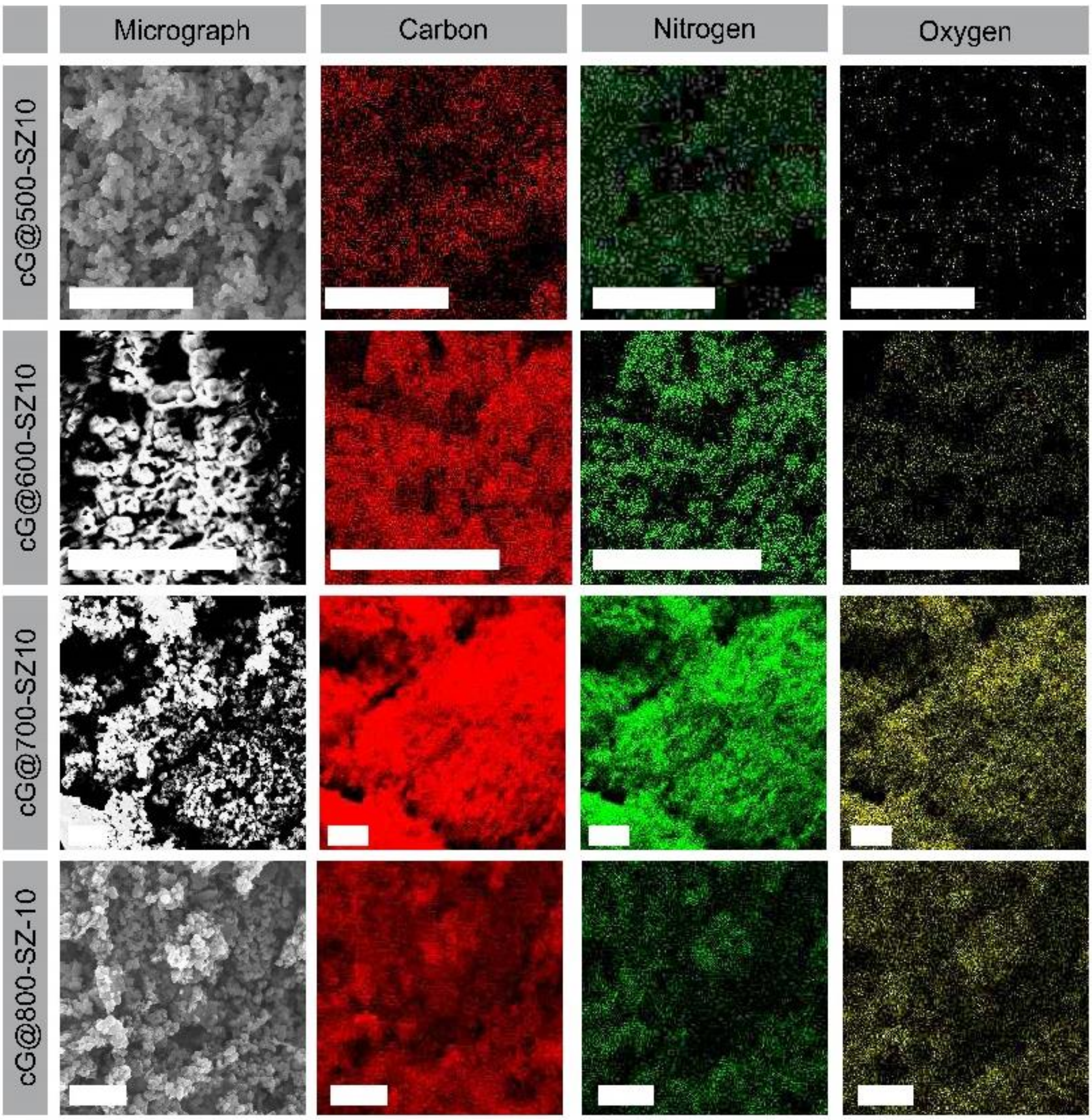

Figure S9. SEM micrographs of guanine condensed at 500 °C (A) and 800 °C (B) with increasing amounts of porogen.

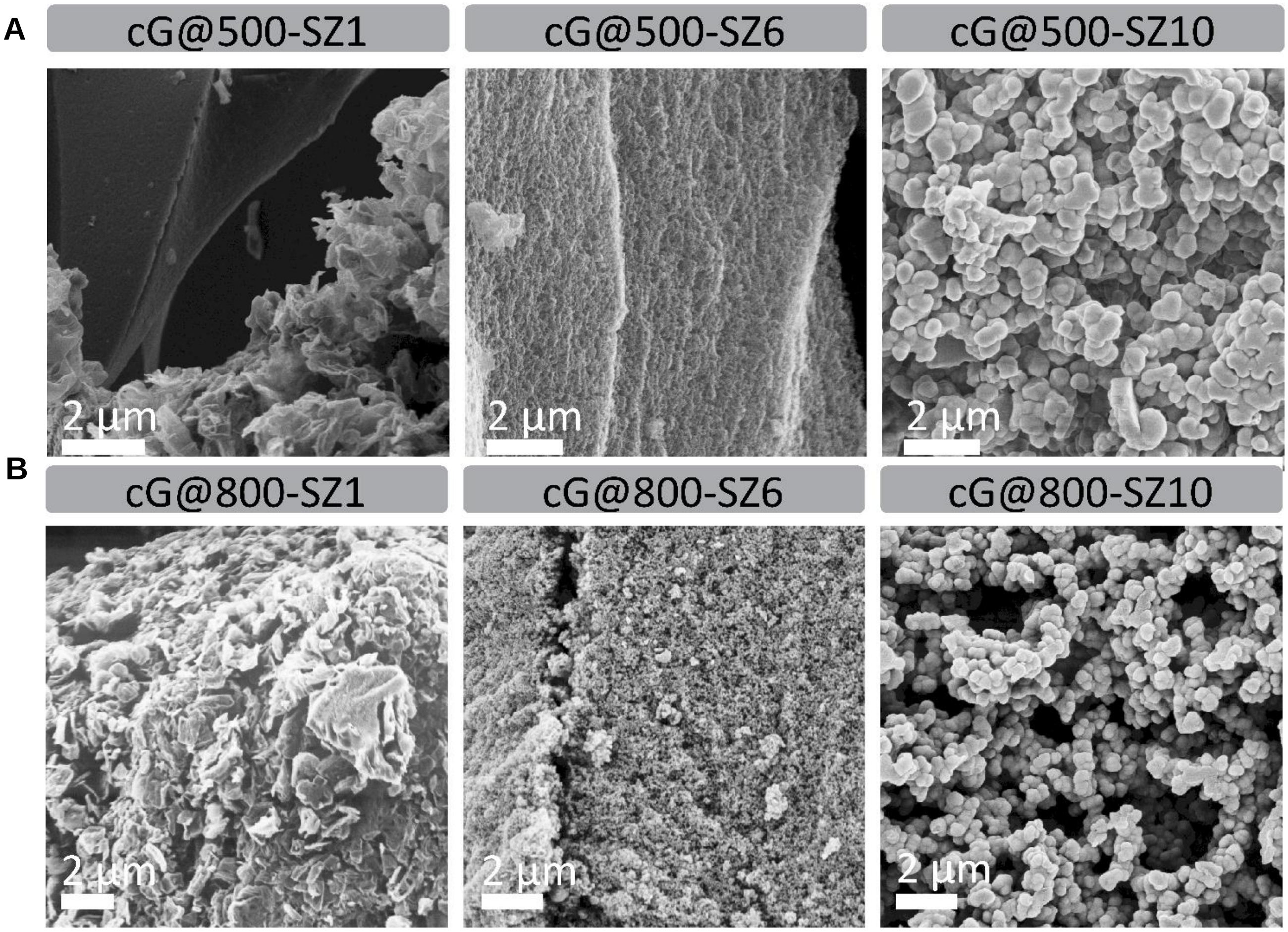

Figure S10. TGA of the mixture of guanine and NZ in a 1:10 ratio.

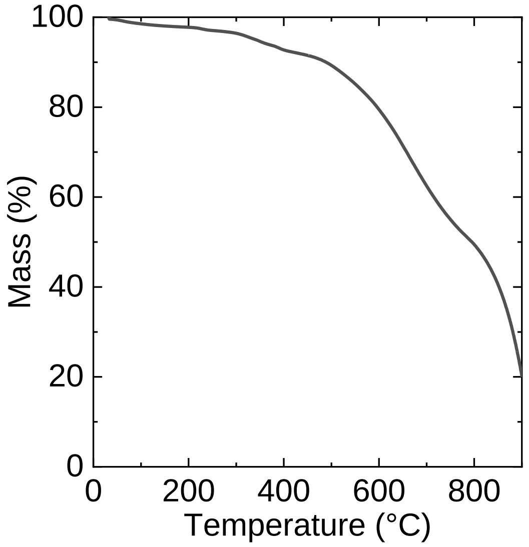

Figure S11. N_2 adsorption/desorption at 77 K (left panel) and CO_2 adsorption/desorption isotherms at 273 K (right panel) of guanine condensed at 500 °C (A and C) and 800 °C (B and D) with an increasing guanine to porogen ratio.

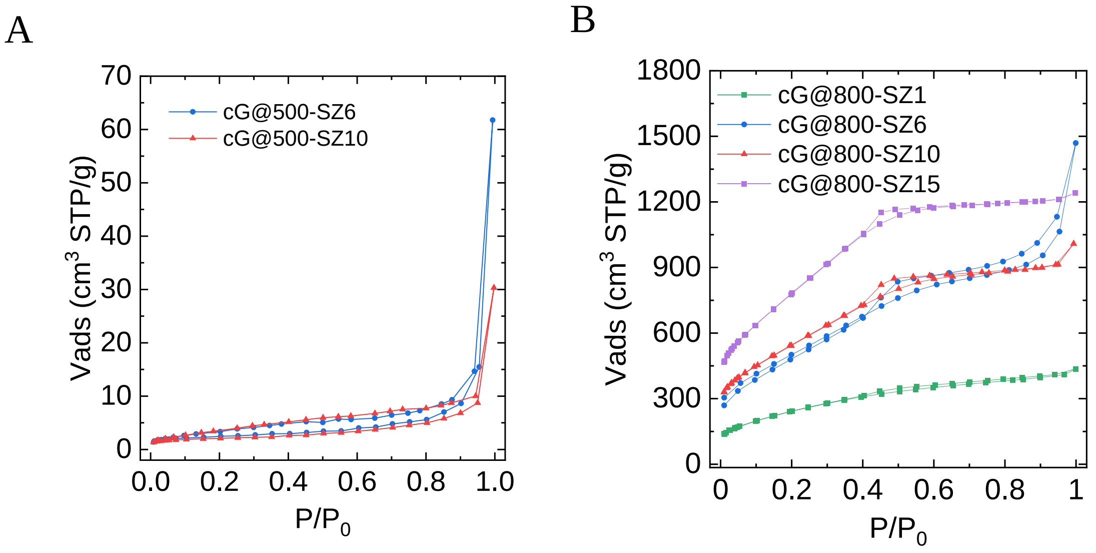

Table S2. Data extracted from N2 physisotption data at 77K and composition according to EDX of cG@800-SZ15.

Sample	S_{BET}	$V_T{}^a$	EDX (at%)		
	m^2g^{-1}	cm^3g^{-1}	C	N	O
cG@800-SZ1	880	0.60	71.4	19.4	3.1
cG@800-SZ6	1894	1.60	78.7	12.9	5.7
cG@800-SZ10	1982	1.42	81.2	11.0	6.7
cG@800-SZ15	2857	1.90	77.3	12.7	10.0

Figure S12. Argon physisorption isotherms (a) and DFT pore size distribution of cG@700-SZ10 (b) and cG@800-SZ10 (c).

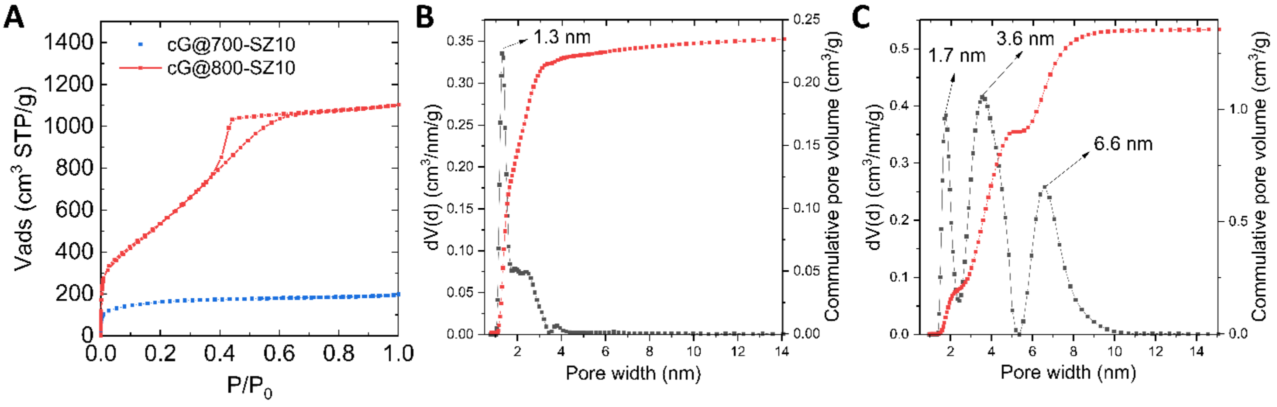

Table S3. Literature overview on Knoevenagel condensation reactions run using nitrogen doped materials as catalysts.

Material	Nitrogen	S_{BET}	Pre-treatment	Reaction compounds	Reaction time / rate	Conversion / selectivity	Ref.
	wt.%	m^2/g		Benzaldehyde +	h / mmol $g^{-1}h^{-1}$	%	
N-doped Carbon	-	625	200 °C, 60 min, argon	Ethylcyanoacetate	20	32.7 / 13.7	1
C_3N_4-TEOS			200 °C, 60 min, argon	Ethylcyanoacetate	20	100 / 50.9	1
BS-700	4.4	4.6	-	Malononitrile	-	2.2 / 100	2
BSNC-600	4.65	962	-	Malononitrile	-	8.3 / 100	2
BSNC-700	2.79	1475	-	Malononitrile	-	16.1 / 100	2
BSNC-700-tBU	2.79	1475	tBU (1.0 M)	Malononitrile	-	79.9 / 100	2
BSNC-800	1.06	2271	-	Malononitrile	-	8.2 / 100	2
MM-NH2	1.6	120	Post functionalized with propargylamine	Malononitrile	20	73 / -	3
FM-NH2	5.9	480	Post functionalized with propargylamine	Malononitrile	20	74 /-	3
MS-NH2	2.1	225	Post functionalized with propargylamine	Malononitrile	20	92 / -	3
FS-NH2	3.5	758	Post functionalized with propargylamine	Malononitrile	20	76/ -	3
HC-10Bu2IMBr	8.22	170	-	Malononitrile	12	98/ -	4
HC-10Bu2IMBr	8.22	170	-	Acetophenon	20	99/ -	4
Mpg-C3N4	-	-	-	Malononitrile	2	33 / 74	5
Mpg-C3N4-tBu	-	-	tBuOk	Malononitrile	2	80 / 96	5
PAN-C500-AO400	17.3 (at.)			Ethyl cyanoacetate	- / 199	- / -	6

References

1. Shcherban, N. D.; Mäki-Arvela, P.; Aho, A.; Sergiienko, S. A.; Yaremov, P. S.; Eränen, K.; Murzin, D. Y., Melamine-derived graphitic carbon nitride as a new effective metal-free catalyst for Knoevenagel condensation of benzaldehyde with ethylcyanoacetate. *Catal.: Sci. Technol.* **2018,** *8* (11), 2928-2937.
2. Mi, B.; Chen, X.; Jiang, C.; Wang, J.; Chen, X.; Zhang, B.; Liu, X.; Liu, Z.; Fei, B., Nitrogen-Doped Porous Carbon Derived from Bamboo Shoot as Solid Base Catalyst for Knoevenagel Condensation and Transesterification Reactions. *Catalysts* **2018,** *8* (6), 232.
3. Kaper, H.; Grandjean, A.; Weidenthaler, C.; Schüth, F.; Goettmann, F., Surface Diels–Alder Reactions as an Effective Method to Synthesize Functional Carbon Materials. *Chem. - Eur. J.* **2012,** *18* (13), 4099-4106.
4. Demir-Cakan, R.; Makowski, P.; Antonietti, M.; Goettmann, F.; Titirici, M. M., Hydrothermal synthesis of imidazole functionalized carbon spheres and their application in catalysis. *Catal. Today* **2010,** *150* (1-2), 115-118.
5. Su, F.; Antonietti, M.; Wang, X., Mpg-C 3N 4 as a solid base catalyst for Knoevenagel condensations and transesterification reactions. *Catal.: Sci. Technol.* **2012,** *2* (5), 1005-1009.
6. Fujita, S.-i.; Katagiri, A.; Watanabe, H.; Asano, S.; Yoshida, H.; Arai, M., Preparation of Nitrogen-Doped Carbon from Polyacrylonitrile and its Application as a Solid-Base Catalyst. *ChemCatChem* **2015,** *7* (18), 2965-2970.
